# Gulls as a host for both gamma and deltacoronaviruses

**DOI:** 10.1038/s41598-023-42241-8

**Published:** 2023-09-13

**Authors:** Katarzyna Domańska-Blicharz, Justyna Miłek-Krupa, Anna Pikuła

**Affiliations:** 1 Department of Poultry Diseases, National Veterinary Research Institute, al. Partyzantow 57, 24-100 Pulawy, Poland; 2 Holy Cross Cancer Center, Stefana Artwińskiego 3, 25-734 Kielce, Poland

**Keywords:** Genetics, Microbiology, Molecular biology

## Abstract

The coronaviruses (CoV) are ubiquitous pathogens found in wide variety of hosts that constantly pose a threat to human and animal health as a result of their enormous capacity to generate genetic changes. Constant monitoring of virus reservoirs can constitute an early-warning tool and control the spread and evolution of the virus. Coronaviruses are common in wild birds, globally, and birds of the *Charadriiformes* in particular have been demonstrated to be carriers of delta- (dCoV) and gammacoronaviruses (gCoV). In this paper, we present the genetic characterisation of five CoV strains from black-headed (*Chroicocephalus ridibundus*) and common (*Larus canus*) gulls. Whole genome sequence analysis showed high similarity of detected dCoV in gulls to previously identified strains from falcon, houbara, pigeon and gulls from Asia (UAE, China). However, phylogenetic analysis revealed bifurcation within a common branch. Furthermore, the accumulation of numerous amino acid changes within the S-protein was demonstrated, indicating further evolution of dCoV within a single gull host. In turn, phylogenetic analysis for the most of the structural and non-structural genes of identified gCoV confirmed that the strain belongs to the duck coronavirus 2714 (DuCoV2714) species within *Igacovirus* subgenera, while for the spike protein it forms a separate branch not closely related to any gCoV species known to date. The current study provides new and significant insights into the evolution and diversification of circulating coronaviruses in members of *Laridae* family.

## Introduction

Coronaviruses have an envelope equipped with protruding structures on the surface called spikes (S) and the genome in the form of a positive-sense single-stranded RNA. RNA viruses tend to mutate at a maximum possible rate close to the error threshold, beyond which mutations can result in virus failure. This threshold is among others defined by genome lengths, which in coronavirus is approximately 27–32 kb. High rate of mutations is mostly attributable to low fidelity of the RNA-dependent RNA polymerase (RdRp) machinery, which has limited ability of proofreading^1^. This causes such viruses to exist as a collection of variants in the host even during a short infection, and some of these variants may provide measurable benefits^2^. These properties of coronaviruses to generate changes and acquire new properties, including host switching, resulted in a worldwide Covid-19 pandemic with more than 670 million infections and almost 7 million deaths (https://www.worldometers.info/coronavirus/)^3^. The *Orthocoronavirinae* subfamily includes four genera of viruses: *Alpha*-, *Beta*-, *Gamma-* and *Deltacoronavirus*. Only gamma- (gCoVs) and deltacoronaviruses (dCoVs) infect birds, but some of them can also infect mammals^4^.

The *Gammacoronavirus* includes three subgenera and 5 species while the *Deltacoronavirus* similarly contains three subgenera but seven species. (https://talk.ictvonline.org/taxonomy/). 

Our recent study revealed the 4.15% prevalence of coronaviruses in wild birds population in Poland and the main reservoirs were birds from orders *Anseriformes* and *Charadriiformes*^5^. Gammacoronaviruses were most often identified with a detection rate of 3.5% in birds of six orders (*Anseriformes, Charadriiformes, Columbiformes*, *Galliformes*, *Gruiformes* and *Passeriformes*)*.* Deltacoronaviruses have been detected in birds from three orders (*Charadriiformes*, *Galliformes* and *Suliformes*) with detection rate of 0.7%. The obtained results from our studies reveal a picture of gulls as a host for both gamma- and deltacoronaviruses, with a clear grouping of these viruses, i.e. in the genus *Gammacoronavirus* among igacoviruses belonging to the DuCoV2714 species and in the genus *Deltacoronavirus* among buldecoviruses belonging to the WeCoV-HKU16 species. Similar results were obtained previously, as both gamma- and deltacoronaviruses were detected in different gulls worldwide^6–10^. Moreover, the whole genome sequence of deltacoronaviruses detected in black-headed gulls (*Chroicocephalus ridibundus*) (BHGdCoV) in Chinese Yunnan Province were also recently described^11^. In contrast, a gammacoronavirus identified in great black-backed gull (*Larus marinus*) (BBGgCoV) in Canada's Newfoundland managed to sequence about 1/3 of the genome, containing a viral polymerase fragment (ORF1a)^12^. In this paper, we report the genome characterization of both genera of coronaviruses detected in different species of gulls in Poland.

## Results

We selected 27 coronaviral RNA positive samples from previous studies^5^ for interrogation. Through NGS, nearly whole genomic sequences were obtained for five virus samples. Among these viruses were four deltacoronaviruses identified in three black-headed gulls (BHGdCoV/Poland/P350/2017, GenBank accession number OQ535492, BHGdCoV/Poland/P052/2018, OQ535490 and BHGdCoV/Poland/P103/2018, OQ535491) and in one common gull (CGdCoV/Poland/P005/2019, OQ535493). Additionally, one sequence of gammacoronavirus detected in common gull (*Larus canus*) (CGgCoV/Poland/P014/2019, OQ535494) was achieved. Genomes with a length of more than 26,000 nt in the case of deltacoronaviruses and about 23,300 nt in the case of gammacoronavirus were obtained. Unfortunately, the resulting gCoV sequence was incomplete, requiring the design and use of several primers to fulfill it, which was abandoned due to the limited amount of obtained sample.

The genome organization of all four dCoVs was similar to Chinese BHGdCoVs (HNU4) as well as to falcon HKU27, houbara HKU28 and pigeon HKU29 dCoVs found in United Arab Emirates and contained 10 genes encoded in 6 open reading frames (ORFs) with the order: 5'UTR-1ab-S-E-M-NS6-N-NS7a-NS7b-NS7c-NS7d-3′UTR. Generally, the length of these genes and transcribed proteins among Polish strains was the same except for the spike (S), matrix (M) and nucleoprotein (N) structures. The Polish dCoV strains differed in the S protein length by 12 nt/4 aa. The longest Spike was present in the BHGdCoV/Poland/P103/2018 strain isolated from the black-headed gull, while the shortest in the CGdCoV/Poland P005/2019 strain isolated from the common gull. In contrast, the M and N structures between dCoVs detected in the common gull and the three detected in the black-headed gulls differed by only 3 nt/1 aa.

**Figure 1:**
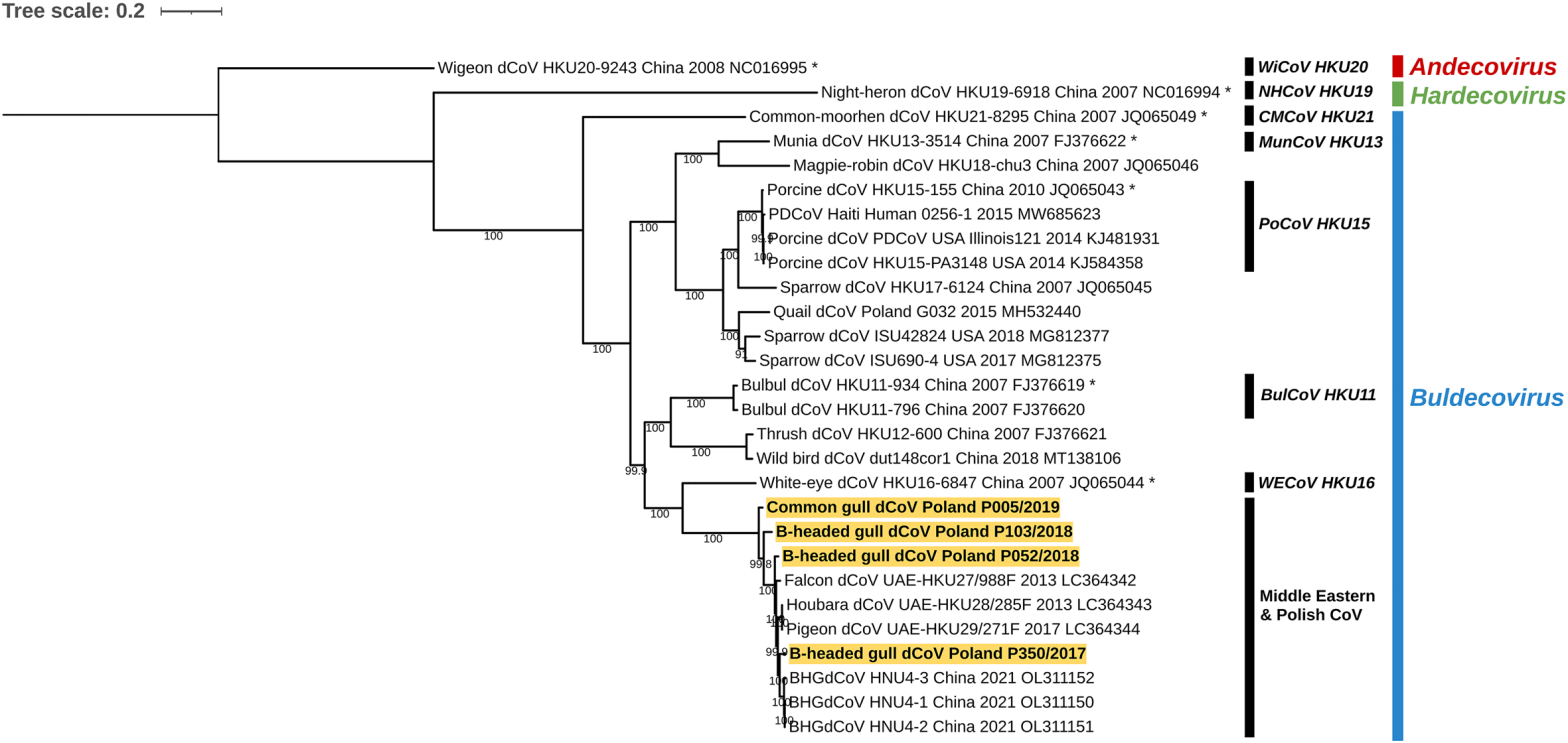
Maximum likelihood phylogenetic tree of the complete genomes of deltacoronaviruses constructed using GTR + F + I + G4 model with 1000 bootstrap iterations. The *Deltacoronavirus* subgenera are denoted with designation and colors, the Polish strains characterized in presented study are bolded and highlighted with yellow.

The phylogeny based on the complete genome sequences revealed that all four deltacoronavirus strains from gulls sampled in Poland formed a monophyletic clade with other deltacoronaviruses from birds identified in the United Arab Emirates and China, close to white-eye dCoV HKU16 strain which is a recognized, designed species within Buldecovirus subgenus (Fig. 1). However, the viruses found in presented study in gulls and those from Chinese gull, as well as from Middle Eastern falcon, houbara and pigeon, appear sister to white-eye dCoV HKU16, but do not fall within the species based on the genetic distance thresholds outlined by the ICTV. They appear to belong to Buldevovirus, but this is not officially ratified. Moreover, the Buldecoviruses from this branch showed further bifurcation. Two strains BHGdCoV/Poland/P350/2017 and BHGdCoV/Poland/P052/2018 clustered together with the HNU4 dCoV strains from Chinese black-headed gulls and dCoVs identified in birds in the United Arab Emirates. The other two Polish dCoV strains BHGdCoV/Poland/P103/2018 and CGdCoV/Poland/P005/2019 seems be distinct. However, more strains would need to be analyzed to be sure if they form separate groups. Sequence analysis of the full genome revealed that Polish dCoV strains shared nucleotide identity of 92.8–96.7% (Supplementary Table S1a). The S protein of Polish strains contained many amino acids altered in comparison to the deltacoronaviruses identified in black-headed gulls in China. More than 30 changes in the NTD domain of the S1 region were found in all Polish virus strains. In contrast, only 3 mutations were found in the CTD domain of the S1 region common to all Polish strains, although one BHGdCoV/Poland/P103/2018 strain contained an additional 37 mutations compared to the others coronaviruses analyzed. The differences in the S2 region were relatively less. Only 5 common mutations were found in all four dCoV strains, and some of them had a few additional changes. However, the BHGdCoV/Poland/P005/2019 strain had the most diverse S2 region, which contained more than 50 aa alterations but also several deletions compared to the other strains (Supplementary Figure S2).The similarity of the other structural proteins of dCoVs from Poland compared to those from China and Middle East was higher than that of the S protein and was 98.8% for the E protein, 94–100% for the M protein, and 97.4–98.5% for the N protein. A number of differences were also found in the amino acid structure of non-structural proteins. The most conserved proteins in Polish, Chinese and Middle Eastern dCoV strains were NS6 (with 98.9–100% homology) and NS7d (with 95.4–100% homology). In contrast, the highest variations in amino acid sequences were observed in the NS7a (92.2–98.2%) and NS7c (94.1–99.2%) proteins (Supplementary Table S1b).

**Figure 2:**
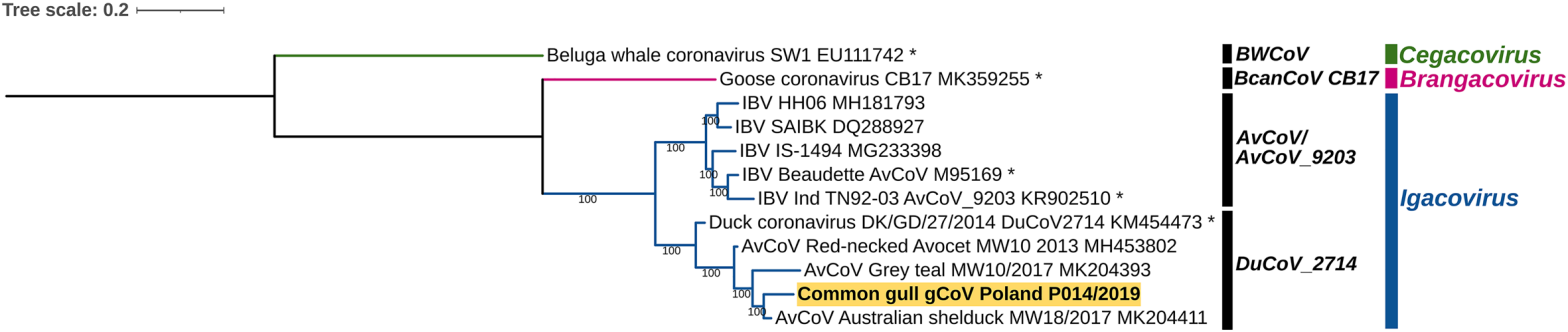
Maximum likelihood phylogenetic analysis of nucleotide sequence of viral replicase (RdRp) of gammacoronaviruses constructed using TIM2 + F + G4 model with 1000 bootstrap iterations. The *Gammacoronavirus* subgenera are denoted with designation and colors, the Polish strain characterized in presented study is bolded and highlighted with yellow.

The obtained partial genome of gammacoronavirus gCoV/CG/Poland/P014/2019 consisted of 23302 nucleotides and contained two ORFs which encode partial polyproteins 1a (pp1a) and whole 1b (pp1b), then ORFs S, E, M, 4b, 4c, 5a, 5b and N (Supplementary Table S3). Closely related sequences genome of gCoV/CG/Poland/P014/2019 were identified using BLASTn algorithm on the server of the National Center for Biotechnology Information (NCBI). The most similar sequences with homology of 97.5% showed Australian gammacoronavirus strain identified in representative of *Charadriiformes*, AvCoV/Ruddy turnstone/MW11_1o/2015 at a length of more than 15,000 nucleotides of the ORF1ab fragment. The similarity of the gammacoronavirus AvCoV/Gull/Canada/B29/2015 detected in gulls was also high at 97.5%, but on a much shorter ORF1a fragment (approximately 7500 nt). Further strains with homology of 86.1–92.5% for a length of about 15,000 nt were gammacoronaviruses belonging to the species of DuCoV2714 identified in such birds as Australian shelducks (*Tadorna tadornoides*), grey teal (*Anas gracilis*), red-necked avocet (*Recurvirostra novaehollandiae*) and domestic duck. The homology to the species of AvCoV/AvCoV9203 was below 82.8%. Phylogenetic analysis of the viral replicase performed for the gCoV strains for which BLAST analysis showed the highest homology to Polish sequence, as well as other representing individual species of the *Gammacoronavirus* genus, revealed that CGgCoV/Poland/P014/2019 was on a common branch formed by viruses of the DuCoV2714 species, distinct from that formed by infectious bronchitis virus (IBV) strains (AvCoV/AvCoV9203) (Fig. 2).

The similarity between the Polish strain and representatives of the *Brangacovirus* and *Cegacovirus* subgenera was 66.0–66.1 and 52.9%, respectively (Supplementary Table S4). The gCoV strain identified in this study shared identity of 68.5–81.4 and 82.3–86.1% to three representatives of DuCoV2714 in their E and M proteins, respectively. Similar identity (68.1–88.3%) was also observed for N protein of available DuCoV2714 strains. Similarities of E and M proteins of detected CGgCoV/Poland/P014/2019 to other representatives of *Igacovirus* and *Brangacovirus* subgenera were 70.6–73.3% and 68.6–71.7%, respectively. In addition, they shared a relatively lower identity (59.9–66.9%) of N protein. The similarity of these structures to their counterparts in cegacoviruses was only 23.8–41.4%. Interestingly, despite the low similarity of structural proteins, the similarity of non-structural proteins, namely 4b but also 5a was higher, in the range of 88.4–94.4%. In contrast, the strain CGgCoV/Poland/P014/2019 shared a significantly lower identity (21.8–36.6%) to other gammacoronaviruses in their S protein. The S protein, containing 1122 amino acids, has an unique S1/S2 cleavage site (520-FLPDHSYFKVTPVYVNYTEYSPLVFED-546) which results in the formation of subdomains S1 (546 aa) and S2 (576 aa). Interestingly, comparison of only the S1 coding region shows a relatively low homology between the Polish CoV strain from gull and the others CoVs at only 13.2–15.6%, while the similarity of the S2 coding region was higher in the range of 41.4–49.6% (Supplementary Figure S5). Molecular analysis showed that all igacoviruses belonging to the three species, DuCoV2714 and AvCoV/AvCoV9203, are particularly different from the newly detected CGgCoV/Poland/P014/2019 in the S gene region, particularly the S1 domain. In contrast, the S2 domain contains more similar nucleotides, particularly within the approximately 219–228 nucleotide fragment (corresponding to 73–76 aa) at the very end of the S2 domain potentially containing two structures: the transmembrane domain and the cytoplasmic tail, whose homology reached 79.4–81.4%. These results are reflected in the phylogenetic analysis performed for the aa sequence of spike protein, where the Polish strain forms a singularly separate branch on the phylogenetic tree (Supplementary Figure S6). However, as analysis showed, there is no evidence of recombination involvement in the emergence of the CGgCoV/Poland/P014/2019 strain with any previously known coronavirus.

## Discussion

In our previous paper, the prevalence of coronaviruses in population of wild birds in Poland was investigated. The study showed that birds of the genus *Charadriiformes* were the main reservoir of both gamma- and deltacoronaviruses. In order to better understand the variability of the virus, including in relation to the host, we have attempted to acquire and molecularly characterize the full coronavirus genome sequences from gulls. As a results, five complete CoV genome sequences were obtained from two species of gulls, the black-headed and common gulls. It should be noted that the whole genome sequences of wild bird coronaviruses available to date are from outside Europe, mainly Asia, the Middle East, Australia or North America^12–19^. The leading group having a major contribution to the identification and characterization of dCoVs based on full genome sequences are Hong Kong scientists studying wild bird populations in China^19,13^. They also detected and characterized dCoVs in three Middle Eastern bird species, falcon (FalCoV UAE-HKU27), houbara (HouCoV UAE-HKU28) and pigeon (PiCoV UAE-HKU29)^14^. Recently, of the 460 samples tested in 2021 from black-headed gulls collected in Yunnan Province, three were dCoV-positive and their whole genomes were obtained. These BHGdCoV genome sequences showed high identity with the genomes of HKU27, HKU28 and HKU29 from Middle East which suggest the close evolutionary relationship and potential interspecies and transboundary transmission of these viruses^11^. To the best of our knowledge, the dCoV whole sequences described in this study are therefore the first from wild birds from Europe (Poland).

The genome organization of all four dCoVs was similar to Chinese BHGdCoVs as well as to falcon HKU27, houbara HKU28 and pigeon HKU29 dCoVs found in United Arab Emirates. The relatedness of these viruses was confirmed by phylogenetic analysis based on the complete genome (Fig. 1), but also for selected structural and non-structural proteins (data not shown). In contrast, the highest variation was observed within the structure of spike protein. The S protein contained many altered amino acids compared to deltacoronaviruses identified in black-headed gulls in China. Interestingly, all mutations that were recently described as new potential host-specific receptors for gulls were not conserved in the Polish dCoVs. Moreover, the amino acid sequence of these sites in the Polish strains was the same as in the Middle Eastern bird strains^14^. Thus, they are unlikely to be responsible for binding to the BHG receptor, as stated before^11^. The Polish dCoVs analyzed in this study were identified over a period of just over 2 years (the earliest BHGdCoV/Poland/P350/2017 in December 2017, the latest CgdCoV/Poland/P005/2019 in January 2019) but in locations 35–40 km apart (Baltic coast near Gdansk city). However, it can be assumed that they came from different populations of gulls, which may explain the observed diversity of identified deltacoronaviruses. Gulls from Yunnan Province infected with almost identical dCoVs were sampled for 3 months in 2021 (January–March) in one location^11^. On the other hand, nearly identical dCoVs from the Middle East originated from birds sampled over a 24-month period with completely different behavior and habits, and interspecies transmission of these viruses was probably the result of a predator–prey relationship^14^. The diversity of dCoVs identified in gulls in our studies resembles that observed among IBV strains. It is known that different IBVs can differ by 20–25% on a genomic scale and up to 50% of the amino acids in the S1 protein^20,21^. Such variability can lead to significant biological differences between strains, and new serotype variants can arise from even a limited number of amino acid changes in the spike protein^22^. It would be interesting to know whether the observed nt/aa changes affect any features of the dCoVs. However, confirmation of this would certainly require in vitro propagation of the virus, which to the authors' knowledge, no one has managed to do so far.

The second coronavirus species detected in our study in gulls is the gammacoronavirus CGgCoV/Poland/P014/2019 of the *Igacovirus* subgenus closely related to the DuCoV2714 from China in 2014. Similar gCoVs have already been identified in wild birds in Australia (Australian shelducks, ruddy turnstone, grey teal and red-necked avocet) and Canada (great black-backed gull). For most of these viruses mainly ORF1ab was determined, only for two (Australian shelduck and gray teal) a longer genome fragment including S gene was determined. It is interesting that the S gene of CGgCoV/Poland/P014/2019 is distantly related to other known gCoVs. The identity of this structure to other DuCoV2714 strains reached only 34–36%, similar to other *Gammacoronavirus* subgenera. Despite this low degree of S protein homology, some fragments indicate common features. At the very end of the S, a domain potentially containing two structures: the transmembrane domain and the cytoplasmic tail, this homology to other igacoviruses was more than 80%. The analyses performed excluded a recombination event in the origin of the identified CGgCoV/Poland/P014/2019 strain. Although the data exclude the involvement of recombination in the evolution of CGgCoV/Poland/P014/2019 such an explanation may emerge as other coronaviruses, in particular their S genes, become known. A similar situation was described in the genesis of AvCoV/AvCoV9203 igacoviruses^23,24^. Turkey coronavirus and IBV also have a close genetic relationship as nucleotide similarity of whole their genomes is about 86%, apart from of the S gene as it shares only 36% identity^23,25^. Moreover, deep molecular studies suggest that European and North-American TCoV strains have different evolutionary pathways in both continents^23,24^.

A picture of gulls as a host for both gamma- and deltacoronaviruses also appears from the obtained results. This may be due to the specificity of this family. *Laridae* counts 102 species of birds, mainly belonging to the subfamily of gulls and terns, found throughout the world. The family is extremely ecologically diverse. Some species are widespread, while some are found in a very limited area. They also differ in the habitat they occupy—some live exclusively in the marine environment, others only in the freshwater environment, while others live in both. They also differ in their ability to migrate—some migrate between continents, while others are sedentary or migrate locally. Gulls also exhibit a variety of behavior in relation to humans or domesticated animals—from frequent direct contact, sharing the same environment, to species with very occasional contact with the human environment. The diet of *Laridae* is also a contributing factor to the occurrence of various viruses. In general, they are omnivorous, although they consume invertebrates and fish most readily; some species eat diseased or dead animals, thus facilitating the direct transmission of various pathogens. In addition, *Laridae* live in colonies with high densities, where contact between infected individuals can easily occur^26^. All these factors may favour the infection of gulls with various coronaviruses, also described in this paper.

Given the continuous evolution of coronaviruses and the fact that porcine deltacoronaviruses, which most likely evolved from avian deltacoronaviruses, can infect humans, continued studies of the animal reservoir should be conducted to broaden our knowledge of the diversity of these viruses^27–29^. In our study, we described the genetic characteristics of both gamma- and deltacoronaviruses identified in *Laridae* in Poland. It cannot be excluded that two different coronavirus genera simultaneously infect the same cell of one organism bird and, in addition, are on a similar replication cycle. Such simultaneous co-infection of Pacific black duck with gamma- and deltacoronavirus was recently reported in Australia^30^. This creates excellent conditions for the recombination event to occur and the emergence of a new one with completely new properties.

## Material and methods

### Samples

In order to obtain the complete genome sequences of samples identified as CoV-positive in our earlier studies (166 positives including 140 gamma- and 26 deltacoronaviruses), selected samples with the highest viral load confirmed by PCR were subjected to Next Generation Sequencing (NGS) using the MiSeq Personal Sequencer platform (Illumina, USA) offered by the Department of Omics Analysis of PIWet-PIB or the commercial service Genomed SA. (Warsaw, Poland). A total of 27 coronavirus-positive field specimens were subjected to NGS attempts. Briefly, samples (cloacal swabs) were treated with TURBO DNase (Life Technologies, USA) and RNase One (Promega, USA) to remove DNA and extracapsid RNA. The isolation of viral RNA from such treated samples was carried out and then retrotranscribed into DNA using a Superscript IV First-Strand cDNA Synthesis Kit (Invitrogen, USA) and the second strand was synthesized with the addition of Klenow polymerase (New England Biolabs, USA). A 300 bp long paired-end DNA library was prepared using a Nextera XT sample preparation kit (Illumina Inc) and sequencing was performed using a MiSeq Reagent kit v3 (Illumina Inc).

### Bioinformatics analysis and phylogeny

Raw reads were de novo assembled into contigs with SPAdes assembler^31^ using Geneious Prime software, v2023.0.4 (Biomatters Ltd., New Zealand). The obtained nucleotide sequence was then compared with other sequences from GenBank database using the BLAST algorithm and those with the highest homology were downloaded for further analysis (http://www.ncbi.nlm.nih.gov/BLAST/). Alignments of nucleotide sequences were performed using the MAFFT method and the percentage of nucleotide and amino acid sequences similarities were assessed in above mentioned software. The alignments were then exported to IQ-TREE software (version 1.6.12) to estimate the best evolutionary model and to infer phylogenetic trees by maximum likelihood algorithm. The confidence levels for the branches were determined by Shimodaira-Hasegawa test and 1000 replicates of bootstrap^32^. The tree visualization was performed using the iTOL v6 online tool^33^. To detect any recombination events, the complete genome of the detected CoVs, and selected the most similar sequences were analyzed using different methods available in the RDP package v.4^34^.

### Ethical approval

The authors declare that they have not conducted any experiments on animals. This paper is only concerned with the sequencing results. Therefore, we do not need to submit any additional statements.

### Supplementary Information

Supplementary Information.

## Data Availability

The complete genome sequences generated in this study were submitted to the GenBank database (https://www.ncbi.nlm.nih.gov/genbank/) under accession numbers OQ535490–OQ535494.

## References

[1] Gorbalenya AE, Enjuanes L, Ziebuhr J, Snijder EJ (2006). Nidovirales: Evolving the largest RNA virus genome. Virus Res..

[2] Lauring AS, Andino R (2010). Quasispecies theory and the behavior of RNA viruses. PLoS Pathog..

[3] Wells HL (2023). The coronavirus recombination pathway. Cell Host Microbe.

[4] Cui J, Li F, Shi Z-L (2019). Origin and evolution of pathogenic coronaviruses. Nat. Rev. Microbiol..

[5] Domanska-Blicharz K, Milek-Krupa J, Pikula A (2021). Diversity of coronaviruses in wild representatives of the aves class in Poland. Viruses-Basel.

[6] Muradrasoli S (2009). Broadly targeted multiprobe QPCR for detection of coronaviruses: Coronavirus is common among mallard ducks (*Anas platyrhynchos*). J. Virol. Methods.

[7] Hepojoki S, Lindh E, Vapalahti O, Huovilainen A (2017). Prevalence and genetic diversity of coronaviruses in wild birds, Finland. Infect. Ecol. Epidemiol..

[8] Wille M, Holmes EC (2020). Wild birds as reservoirs for diverse and abundant gamma- and deltacoronaviruses. FEMS Microbiol. Rev..

[9] Marchenko V (2022). Diversity of gammacoronaviruses and deltacoronaviruses in wild birds and poultry in Russia. Sci. Rep..

[10] Zhigailov AV (2022). Prevalence and genetic diversity of coronaviruses, astroviruses and paramyxoviruses in wild birds in southeastern Kazakhstan. Heliyon.

[11] Chu K-K (2022). Characterization of deltacoronavirus in black-headed gulls (*Chroicocephalus ridibundus*) in South China indicating frequent interspecies transmission of the virus in birds. Front. Microbiol..

[12] Canuti M (2019). Discovery and characterization of novel RNA viruses in aquatic north american wild birds. Viruses.

[13] Woo PCY (2009). Comparative analysis of complete genome sequences of three avian coronaviruses reveals a novel group 3c coronavirus. J. Virol..

[14] Lau SKP (2018). Discovery and sequence analysis of four deltacoronaviruses from birds in the Middle East reveal interspecies jumping with recombination as a potential mechanism for avian-to-avian and avian-to-mammalian transmission. J. Virol..

[15] Zhu W (2021). Genomic characterization of a new coronavirus from migratory birds in Jiangxi Province of China. Virol. Sin..

[16] Papineau A (2019). Genome organization of Canada goose coronavirus, a novel species identified in a mass die-off of Canada Geese. Sci. Rep..

[17] Wille M, Shi M, Klaassen M, Hurt AC, Holmes EC (2019). Virome heterogeneity and connectivity in waterfowl and shorebird communities. ISME J..

[18] Wille M, Shi M, Hurt AC, Klaassen M, Holmes EC (2021). RNA virome abundance and diversity is associated with host age in a bird species. Virology.

[19] Woo PCY (2012). Discovery of seven novel mammalian and avian coronaviruses in the genus deltacoronavirus supports bat coronaviruses as the gene source of alphacoronavirus and betacoronavirus and avian coronaviruses as the gene source of gammacoronavirus and deltacoronavirus. J. Virol..

[20] Cavanagh D (2005). Coronaviruses in poultry and other birds. Avian Pathol..

[21] Cavanagh D, Gelb J, Saif YM (2008). Diseases of Poultry.

[22] Valastro V (2016). S1 gene-based phylogeny of infectious bronchitis virus: An attempt to harmonize virus classification. Infect. Genet. Evol..

[23] Brown PA (2016). First complete genome sequence of European turkey coronavirus suggests complex recombination history related with US turkey and guinea fowl coronaviruses. J. Gen. Virol..

[24] Jackwood MW (2010). Emergence of a group 3 coronavirus through recombination. Virology.

[25] Jackwood MW, Hall D, Handel A (2012). Molecular evolution and emergence of avian gammacoronaviruses. Infect. Genet. Evol..

[26] Arnal A (2015). Laridae: A neglected reservoir that could play a major role in avian influenza virus epidemiological dynamics. Crit. Rev. Microbiol..

[27] Chen Q (2018). The emergence of novel sparrow deltacoronaviruses in the United States more closely related to porcine deltacoronaviruses than sparrow deltacoronavirus HKU17. Emerg. Microbes Infect..

[28] Boley PA (2020). Porcine deltacoronavirus infection and transmission in poultry, United States. Emerg. Infect. Dis..

[29] Lednicky JA (2021). Independent infections of porcine deltacoronavirus among Haitian children. Nature.

[30] Vibin J (2018). Metagenomics detection and characterisation of viruses in faecal samples from Australian wild birds. Sci. Rep..

[31] Bankevich A (2012). SPAdes: A new genome assembly algorithm and its applications to single-cell sequencing. J. Comput. Biol..

[32] Nguyen LT, Schmidt HA, von Haeseler A, Minh BQ (2015). IQ-TREE: A fast and effective stochastic algorithm for estimating maximum-likelihood phylogenies. Mol. Biol. Evol..

[33] Letunic I, Bork P (2021). Interactive Tree Of Life (iTOL) v5: An online tool for phylogenetic tree display and annotation. Nucleic Acids Res..

[34] Martin DP, Murrell B, Golden M, Khoosal A, Muhire B (2015). RDP4: Detection and analysis of recombination patterns in virus genomes. Virus Evol..

