# Supplementary material for: Gulls as a host for both gamma and deltacoronaviruses
Source: Sci Rep. 2023 Sep 13;13:15104. doi: 10.1038/s41598-023-42241-8 (PMC10499781; doi:10.1038/s41598-023-42241-8)
Supplement: Supplementary file 1 — Supplementary Information. [file 41598_2023_42241_MOESM1_ESM.docx]

Gulls as a host for both gamma and deltacoronaviruses

Katarzyna Domańska-Blicharz, Justyna Miłek-Krupa and Anna Pikuła

Department of Poultry Diseases, National Veterinary Research Institute, al. Partyzantow 57, 24-100 Pulawy, Poland

Correspondence: domanska@piwet.pulawy.pl (K.D.B.)

SUPPLEMMENTARY INFORMATION

Supplementary Table S1

a. Comparison of the nucleotide (whole genome) and amino acid identity of structural proteins* of identified deltacoronaviruses in gulls.

|  | **P350/2017** | | | | | | **P052/2018** | | | | | | **P103/2018** | | | | | | **P005/2019** | | | | | |
| --- | --- | --- | --- | --- | --- | --- | --- | --- | --- | --- | --- | --- | --- | --- | --- | --- | --- | --- | --- | --- | --- | --- | --- | --- |
|  | ORF1ab | S | E | M | N | Whole genome | ORF1ab | S | E | M | N | Whole genome | ORF1ab | S | E | M | N | Whole genome | ORF1ab | S | E | M | N | Whole genome |
| **P350/2017** | - | - | - | - | - | - | 97.1 | 92.9 | 97.6 | 94.5 | 99.4 | 96.7 | 93.9 | 87.7 | 97.6 | 95 | 96.8 | 92.9 | 94.1 | 84 | 100 | 94.5 | 97.4 | 92.8 |
| **P052/2018** | 97.1 | 92.9 | 97.6 | 94.5 | 99.4 | **96.7** | - | - | - | - | - | - | 93.7 | 91.7 | 100 | 98.6 | 97.4 | **93.6** | 93.8 | 89.3 | 97.6 | 95.9 | 98 | **93.6** |
| **P103/2018** | 93.9 | 87.7 | 97.6 | 95 | 96.8 | 92.9 | 93.7 | 91.7 | 100 | 98.6 | 97.4 | 93.6 | - | - | - | - | - | - | 97.1 | 83.2 | 97.6 | 97.2 | 98.8 | 95.1 |
| **P005/2019** | 94.1 | 84.0 | 100 | 94.5 | 97.4 | 92.8 | 93.8 | 89.3 | 97.6 | 95.9 | 98 | 93.6 | 97.1 | 83.2 | 97.6 | 97.2 | 98.8 | 95.1 | - | - | - | - | - | - |
| **HKU27-988F** | 96.5 | 93 | 98.8 | 94 | 97.4 | **96.7** | 96.7 | 96.9 | 98.8 | 99.1 | 98 | **97.4** | 93.7 | 90.1 | 98.8 | 98.2 | 96.5 | 93.5 | 93.8 | 87.6 | 98.8 | 95.4 | 97.1 | 93.3 |
| **HKU28-285F** | 96.7 | 94 | 98.8 | 94 | 98 | **96.5** | 96.6 | 94.3 | 98.8 | 99.5 | 98.5 | **96.9** | 93.9 | 88.6 | 98.8 | 98.2 | 97.1 | 93.3 | 94 | 85 | 98.8 | 95.4 | 97.7 | 93.0 |
| **HKU29-271F** | 96.7 | 93.9 | 98.8 | 94.5 | 98 | **96.7** | 96.6 | 94.2 | 98.8 | 100 | 98.5 | **96.9** | 93.9 | 88.5 | 98.8 | 98.6 | 97.1 | 93.3 | 94 | 84.9 | 98.8 | 95.4 | 97.7 | 93.0 |
| **HNU4-1** | 97 | 95.2 | 98.8 | 95 | 97.7 | **96.9** | 96.5 | 93.7 | 98.8 | 98.2 | 98.3 | **96.8** | 93.9 | 88 | 98.8 | 98.2 | 96.5 | 93.2 | 94.1 | 84.3 | 98.8 | 95.9 | 97.1 | 93.0 |
| **HNU4-2** | 97 | 95.2 | 98.8 | 95 | 97.7 | **96.9** | 96.5 | 93.7 | 98.8 | 98.2 | 98.3 | **96.8** | 93.9 | 88 | 98.8 | 98.2 | 96.5 | 93.2 | 94.1 | 84.3 | 98.8 | 95.9 | 97.1 | 93.0 |
| **HNU4-3** | 96.4 | 95.1 | 98.8 | 95 | 97.7 | **96.7** | 96.2 | 93.8 | 98.8 | 98.2 | 98.3 | **96.7** | 93.8 | 88.1 | 98.8 | 98.2 | 96.5 | 93.2 | 94 | 84.3 | 98.8 | 95.9 | 97.1 | 92.9 |
| **HKU17-6124** | 59.5 | 67.6 | 76.8 | 75.2 | 74.8 | 65.3 | 59.6 | 67.7 | 76.8 | 78 | 75.4 | 65.4 | 59.4 | 67 | 76.8 | 77.1 | 75.1 | 65.8 | 60 | 62.9 | 76.8 | 77.5 | 75.4 | 65.3 |
| **HKU13** | 58.2 | 45.3 | 86.6 | 82.6 | 72 | 64.1 | 58.3 | 45.3 | 86.6 | 81.7 | 72.2 | 64.1 | 58.5 | 45.5 | 86.6 | 82.6 | 72 | 64.4 | 58.6 | 49.3 | 86.6 | 81.7 | 71.8 | 64.3 |
| **HKU16-6847** | 68.6 | 45 | 90.2 | 93.1 | 83.3 | 72.8 | 68.5 | 45 | 92.7 | 94.5 | 83.6 | 72.8 | 68 | 44.8 | 92.7 | 95.4 | 83.6 | 72.8 | 68.2 | 50.2 | 90.2 | 94 | 83.3 | 73.0 |
| **HKU21-8295** | 55.5 | 44.6 | 58.5 | 56 | 60.6 | 62.9 | 55.4 | 45 | 59.8 | 56.4 | 60.1 | 62.9 | 55.1 | 45.2 | 59.8 | 56.4 | 60.2 | 63.1 | 55.2 | 47.2 | 58.5 | 57.2 | 59.6 | 63.0 |
| **HKU15-155** | 59.3 | 44.2 | 75.9 | 77.1 | 73.6 | 64.7 | 59.3 | 44.7 | 75.9 | 77.5 | 74.2 | 64.8 | 59.4 | 44.2 | 75.9 | 77.5 | 73.7 | 65.3 | 59.8 | 48.8 | 75.9 | 78.4 | 74 | 65.4 |
| **HumanPdCoV** | 59.4 | 44.1 | 75.9 | 77.1 | 73.6 | 64.7 | 59.3 | 44.8 | 75.9 | 77.5 | 74.2 | 64.7 | 59.5 | 44.2 | 75.9 | 77.5 | 73.7 | 65.3 | 59.8 | 48.9 | 75.9 | 78.4 | 74 | 65.3 |
| **HKU11-796** | 65.3 | 44.1 | 80.5 | 10.5 | 71.9 | 68.8 | 65.3 | 44.4 | 81.7 | 10.9 | 71.9 | 68.7 | 65 | 44.3 | 81.7 | 10.5 | 72 | 69.0 | 65.3 | 48.7 | 80.5 | 10.5 | 71.7 | 69.2 |
| **G032/2015** | 59.2 | 43 | 77.1 | 71.1 | 72.2 | 65.1 | 59.2 | 43.5 | 77.1 | 72.9 | 72.8 | 65.1 | 59.3 | 43 | 77.1 | 73.9 | 72.3 | 65.7 | 59.5 | 48.4 | 77.1 | 73.4 | 72.5 | 65.7 |

*Percentage of residues which are identical (%) was completed using a MAFFT alignment method.

b. Comparison of amino acid identity of non-structural proteins* of identified deltacoronaviruses in gulls.

|  | **P350/2017** | | | | | **P052/2018** | | | | | **P103/2018** | | | | | **P005/2019** | | | | |
| --- | --- | --- | --- | --- | --- | --- | --- | --- | --- | --- | --- | --- | --- | --- | --- | --- | --- | --- | --- | --- |
|  | **NS6** | **NS7a** | **NS7b** | **NS7c** | **NS7d** | **NS6** | **NS7a** | **NS7b** | **NS7c** | **NS7d** | **NS6** | **NS7a** | **NS7b** | **NS7c** | **NS7d** | **NS6** | **NS7a** | **NS7b** | **NS7c** | **NS7d** |
| **P350/2017** | - | - | - | - | - | 100 | 98.2 | 97.1 | 95.2 | 98.5 | 97.8 | 89 | 92.6 | 92.8 | 98.5 | 97.8 | 90.4 | 91.2 | 93.6 | 96.9 |
| **P052/2018** | 100 | 98.2 | 97.1 | 95.2 | 98.5 | - | - | - | - | - | 97.8 | 89.9 | 95.6 | 96 | 96.9 | 97.8 | 90.8 | 94.1 | 93.6 | 95.4 |
| **P103/2018** | 97.8 | 89 | 92.6 | 92.8 | 98.5 | 97.8 | 89.9 | 95.6 | 96 | 96.9 | - | - | - | - | - | 100 | 95.4 | 95.6 | 96 | 98.5 |
| **P005/2019** | 97.8 | 90.4 | 91.2 | 93.6 | 96.9 | 97.8 | 90.8 | 94.1 | 92 | 95.4 | 100 | 90.8 | 95.6 | 96 | 98.5 | - | - | - | - | - |
| **HKU27-988F** | 98.9 | 92.7 | 97.1 | 98.4 | 98.5 | 98.9 | 93.6 | 100 | 95.2 | 96.9 | 96.8 | 93.1 | 95.6 | 92.8 | 96.9 | 96.8 | 94 | 94.1 | 93.6 | 95.4 |
| **HKU28-285F** | 100 | 92.2 | 97.1 | 96 | 98.5 | 100 | 93.1 | 100 | 99.2 | 96.9 | 97.8 | 92.2 | 95.6 | 95.2 | 96.9 | 97.8 | 93.1 | 94.1 | 91.2 | 95.4 |
| **HKU29-271F** | 100 | 92.2 | 97.1 | 96 | 96.9 | 100 | 93.1 | 100 | 99.2 | 95.4 | 97.8 | 92.2 | 95.6 | 95.2 | 95.4 | 97.8 | 93.1 | 94.1 | 91.2 | 93.8 |
| **HNU4-1** | 100 | 92.7 | 94.1 | 94.4 | 98.5 | 100 | 93.6 | 97.1 | 99.2 | 96.9 | 97.8 | 93.1 | 95.6 | 95.2 | 96.9 | 97.8 | 92.2 | 94.1 | 91.2 | 95.4 |
| **HNU4-2** | 100 | 92.7 | 94.1 | 94.4 | 100 | 100 | 93.6 | 97.1 | 99.2 | 98.5 | 97.8 | 93.1 | 95.6 | 95.2 | 98.5 | 97.8 | 92.2 | 94.1 | 91.2 | 96.9 |
| **HNU4-3** | 100 | 92.7 | 92.6 | 94.4 | 100 | 100 | 93.6 | 95.6 | 99.2 | 98.5 | 97.8 | 93.7 | 92.6 | 95.2 | 98.5 | 97.8 | 92.7 | 91.2 | 91.2 | 96.9 |
| **HKU17-6124** | 71 | 52.5 | 63.6 | n/p | n/p | 71 | 53 | 63.6 | n/p | n/p | 72 | 52.5 | 63.6 | n/p | n/p | 72 | 52.5 | 63.6 | n/p | n/p |
| **HKU13** | 62.4 | 47 | 14,7 | 42.2 | n/p | 62.4 | 47.5 | 14.7 | 41.5 | n/p | 63.4 | 48.4 | 14.7 | 42.2 | n/p | 63.4 | 47 | 14.7 | 42.2 | n/p |
| **HKU16-6847** | 78.5 | 60.3 | 41.2 | n/p | n/p | 78.5 | 60.7 | 41.2 | n/p | n/p | 80.6 | 62.6 | 41.2 | n/p | n/p | 80.6 | 61.2 | 41.2 | n/p | n/p |
| **HKU21-8295** | 25.9 | 34.8 | 14.9 | 46.5 | n/p | 25.9 | 34.8 | 13.4 | 45 | n/p | 25.9 | 32.6 | 13.4 | 45.7 | n/p | 25.9 | 31.7 | 16.4 | 47.3 | n/p |
| **HKU15-155** | 66.7 | 53.9 | 60 | n/p | n/p | 66.7 | 54.3 | 60 | n/p | n/p | 67.7 | 52.5 | 60 | n/p | n/p | 67.7 | 52.5 | 60 | n/p | n/p |
| **HumanPdCoV** | 66.7 | 53.9 | 54.5 | n/p | n/p | 66.7 | 54.3 | 54.5 | n/p | n/p | 67.7 | 53.4 | 54.5 | n/p | n/p | 67.7 | 53.4 | 54.5 | n/p | n/p |
| **HKU11-796** | 68.8 | 47.3 | 20 | 47.3 | n/p | 68.8 | 46.8 | 21.3 | 48 | n/p | 69.9 | 49.5 | 20 | 47.3 | n/p | 69.9 | 49.5 | 21.3 | 47.3 | n/p |
| **G032/2015** | 67.7 | 50.2 | 63.6 | n/p | n/p | 67.7 | 50.2 | 63.6 | n/p | n/p | 68.8 | 48.9 | 63.6 | n/p | n/p | 68.8 | 49.8 | 63.6 | n/p | n/p |

*Percentage of residues which are identical (%) was completed using a MAFFT alignment method, n/p – not present.

HKU27 988F - Falcon dCoV, UAE (LC364342); HKU29 271F - Pigeon dCoV, UAE (LC364344); HKU28 285F - Houbara dCoV, UAE (LC364343); HNU4-1 – black-headed dCoV, China (OL311150); HNU4-2 – black-headed dCoV, China (OL311151); HNU4-3 – black-headed dCoV, China (OL311152), HKU11-796 - bulbul dCoV strain (FJ376620); HKU13-3514 munia dCoV strain - (FJ376622); HKU15-155 - PDCoV strain (JQ065043); HKU16-6847 - white-eye dCoV strain (JQ065044); HKU17-6124 - sparrow dCoV strain (JQ065045); HKU21-8295 - common moorhen dCoV strain (JQ065049); G032/2015 – quail dCoV, Poland (MH532440), HumanPdCoV - PDCoV/Haiti/Human/0256-1/2015 (MW685623).


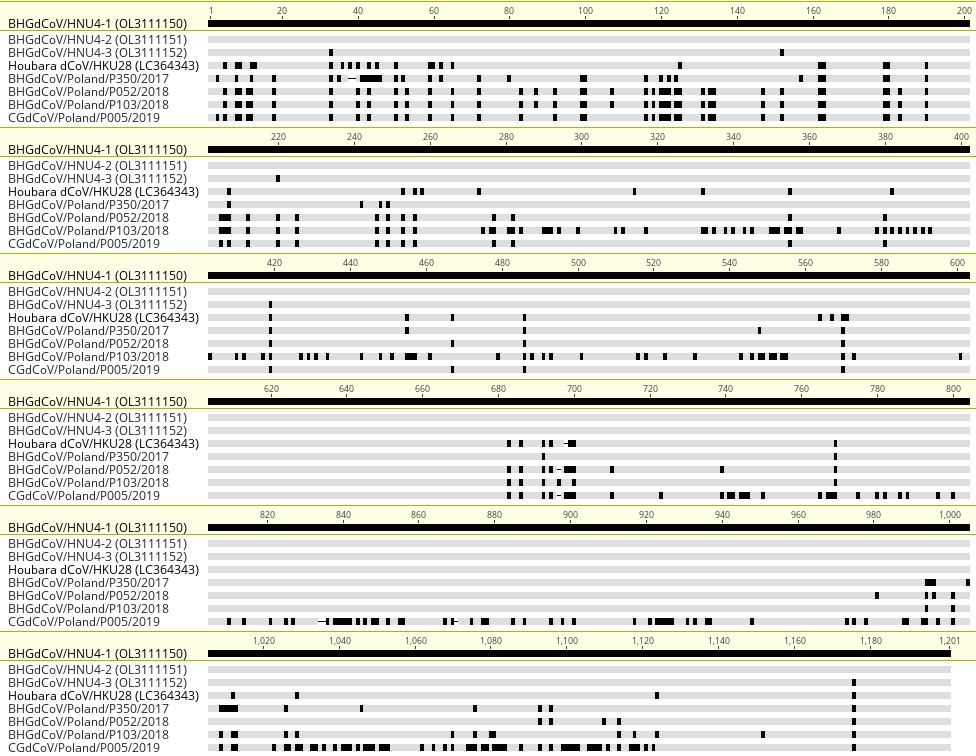


Supplementary Figure S2. Comparison of S protein of Polish deltacoronavirus strains with Chinese counterparts and HKU28 strain. The HKU-1 isolate set as a reference stain and all differences from it are marked as black bars. NTD and CTD domains in S1 is shadowed, The cleavage site between S1 and S2 is indicated with black vertical line.

Supplementary Table S3. Region/gene positions and lengths (nt and aa) deduced from the obtained partial genome sequence of the CGgCoV/Poland/P014/2019 strain.

| **Region/gene** | **5’UTR** | **ORF1a** | **ORF1b** | **S** | **E** | **M** | **4b** | **4c** | **5a** | **5b** | **N** | **3’UTR** |
| --- | --- | --- | --- | --- | --- | --- | --- | --- | --- | --- | --- | --- |
| **Position** | n/a | -8592 | 8665-16658 | 16594-19962 | 19972-20274 | 20271-20942 | 20943-21227 | 21127-21372 | 21369-21566 | 21563-21808 | 21742-22989 | n/a |
| **Length (nt)** | n/a | 8403 | 7992 | 3369 | 303 | 672 | 285 | 246 | 198 | 246 | 1248 | n/a |
| **Length (aa)** | - | 2800 | 2663 | 1122 | 100 | 223 | 94 | 81 | 65 | 81 | 415 | - |

Supplementary Table S4. Comparison of nucleotide (ORF1ab & whole genome) and amino acid identity of structural and nonstructural proteins* of identified gammacoronavirus in gull.

|  | **P014/2019** | | | | | | | | | |
| --- | --- | --- | --- | --- | --- | --- | --- | --- | --- | --- |
|  | **ORF1ab** | **S** | **E** | **M** | **4b** | **4c** | **5a** | **5b** | **N** | **Whole genome** |
| **Ruddy Turnstone CoV** | 97.0 | n/a | n/a | n/a | n/a | n/a | n/a | n/a | n/a | n/a |
| **Gull CoV B29** | 97.5 | n/a | n/a | n/a | n/a | n/a | n/a | n/a | n/a | n/a |
| **Australian Shelduck CoV** | 91.6 | 36.6 | 81.2 | 86.1 | 93.9 | 88.6 | 88.4 | 92.5 | 88.3 | 80.4 |
| **Grey Teal CoV** | 84.6 | 34.8 | 81.4 | 85.0 | n/a | n/a | n/a | n/a | 68.1 | 71.6 |
| **Red-Necked Avocet CoV** | 90.1 | n/a | n/a | n/a | n/a | n/a | n/a | n/a | n/a | 69.1 |
| **DuCoV 2714** | 82.7 | 31.3 | 68.5 | 82.3 | n/a | n/a | 90.3 | 94.4 | 82.7 | 74.8 |
| **AvCoV 9203 - IBV** | 72.6 | 34.1 | 73.3 | 71.7 | n/a | n/a | 43.8 | 51.8 | 66.8 | 66.1 |
| **AvCoV - IBV** | 74.1 | 34.9 | 73.3 | 70.8 | n/a | n/a | 45.3 | 49.4 | 66.0 | 66.7 |
| **IBV IS-1494** | 74.3 | 34.9 | 70.6 | 68.6 | n/a | n/a | 48.4 | 49.4 | 66.9 | 66.8 |
| **AFCoV NCN2** | 66.1 | 33.8 | 72.6 | 71.5 | n/a | n/a | n/a | n/a | 59.9 | 58.0 |
| **GoCoV CB17** | 66.0 | 33.9 | 72.9 | 71.2 | n/a | n/a | n/a | 11.5 | 60.4 | 57.9 |
| **Beluga SW1** | 52.9 | 21.8 | 31.1 | 23.8 | n/a | n/a | 9.8 | 11.8 | 41.4 | 41.7 |

*Percentage of residues which are identical (%) was completed using a MAFFT alignment method.

Ruddy Turnstone CoV – Australia/MW11_1o/2015 (MT993597), Gull CoV B29 – Australia/great black-backed gull/B29/2015 (MN175554), Australian Shelduck CoV – Australia/MW18/2017 (MK204411), Grey Teal CoV – Australia/MW10/2017 (MK204393), Red-Necked Avocet CoV – Australia/MW10/2013 (MH453802), DuCoV 2714 – China/DK/GD/27/2014 (NC048214), AvCoV 9203 IBV - Ind TN92-03 (NC048213), AvCoV IBV - Beaudette (NC001451) IBV IS-1494 – Iran/IS-1493 like strain/2015 (MG233398), AFCoV NCN2 – Bean goose (Anser fabialis) CoV/China/NCN2/2019 (MW436465), GoCoV CB17 – Canada goose CoV/Canada/Cambridge Bay/2017 (MK359255), Beluga SW1 – Beluga Whale SW1 CoV (EU111742)


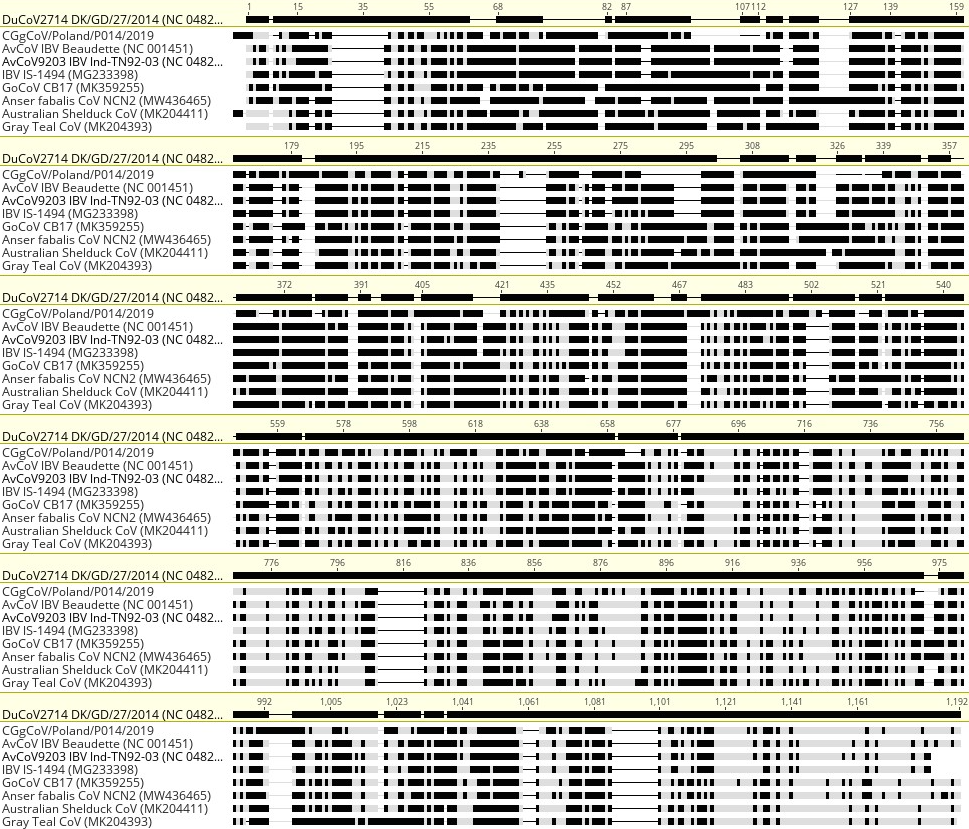


Supplementary Figure S5. Comparison of S protein of Polish gammacoronavirus strain with other available representative of avian *Gammacoronavirus* genus (*Brangacovirus* and *Igacovirus* subgenera). The DuCoV2714 isolate set as a reference stain and all differences from it are marked as black bars. The cleavage site between S1 and S2 is indicated with black vertical line.


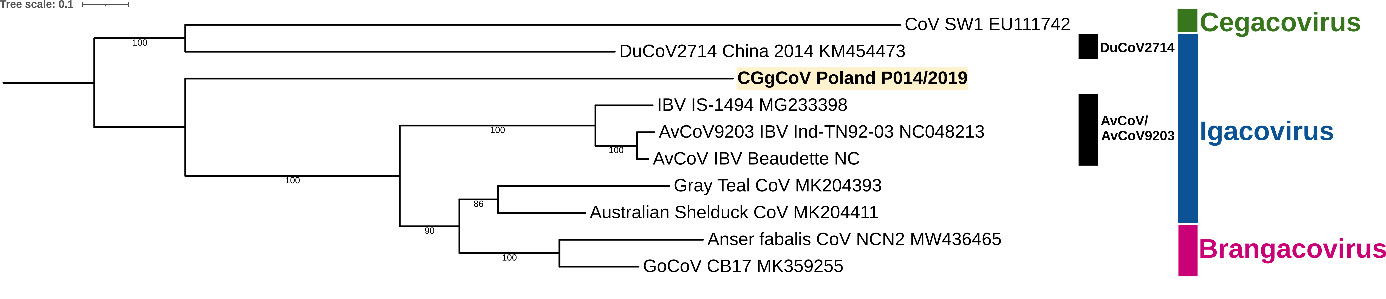


Supplementary Figure S6. Maximum Likelihood phylogenetic analysis of amino acid sequence of spike protein (S) of gammacoronaviruses. Tree was constructed using WAG+F+G4 model with 1000 bootstrap value. The *Gammacoronavirus* subgenera are denoted with designations and colors, the taxa name of the Polish strain is bolded and highlighted with yellow.
